# *In-situ* epitaxial growth of graphene/h-BN van der Waals heterostructures by molecular beam epitaxy

**DOI:** 10.1038/srep14760

**Published:** 2015-10-07

**Authors:** Zheng Zuo, Zhongguang Xu, Renjing Zheng, Alireza Khanaki, Jian-Guo Zheng, Jianlin Liu

**Affiliations:** 1 Quantum Structures Laboratory, Department of Electrical and Computer Engineering, University of California, Riverside, California 92521, USA; 2 Irvine Materials Research Institute University of California Irvine, CA 92697-2800, USA

## Abstract

Van der Waals materials have received a great deal of attention for their exceptional layered structures and exotic properties, which can open up various device applications in nanoelectronics. However, *in situ* epitaxial growth of dissimilar van der Waals materials remains challenging. Here we demonstrate a solution for fabricating van der Waals heterostructures. Graphene/hexagonal boron nitride (h-BN) heterostructures were synthesized on cobalt substrates by using molecular beam epitaxy. Various characterizations were carried out to evaluate the heterostructures. Wafer-scale heterostructures consisting of single-layer/bilayer graphene and multilayer h-BN were achieved. The mismatch angle between graphene and h-BN is below 1°.

Van der Waals (vdW) materials are layered structures bonded with vdW forces. This group of materials have received high interest recently for their novel properties and high potential in various device applications^1^,^2^,^3^. While graphene is one of the most prominent vdW members, beyond graphene materials such as MoS_2_, ZnSe, and hexagonal boron nitride (h-BN) are also being eagerly investigated^4^,^5^,^6^,^7^,^8^,^9^,^10^,^11^. For exploring new paradigm in the two dimensional (2D) devices, atomic-scale heterostructures, which are made from a combination of alternating layers of graphene, h-BN, MoS_2_ and so on, have been paid a great deal of attention. Such heterostructures provide a platform to investigate novel phenomenon in fundamental physics, and there are reports indicating superior properties for device applications^12^,^13^,^14^,^15^. Among these vdW heterostructures, the stacking of h-BN with another vdW layer, in particular, graphene^16^,^17^, is of imminent interest. H-BN has a hexagonal crystal structure similar to graphene’s with less than 2% lattice mismatch^18^. H-BN is a dielectric with a dielectric constant of about 4^19^ and a wide bandgap of ~5.9 eV^7^. H-BN also has exceptional thermal and chemical stabilities^10^. These properties enable h-BN as an excellent chemical and electrical barrier material for graphene and other vdW materials.

To obtain such heterostructures, mechanical exfoliation and chemical vapor deposition (CVD) growth were widely used^20^,^21^,^22^. Much success has been achieved. Nevertheless, direct deposition of high-quality, wafer-scale vdW heterostructures remains challenging. Therefore, non-CVD approaches have been proposed recently, for example, a plasma-assisted deposition method has been used to achieve epitaxial growth of single-domain graphene on h-BN^23^. As a versatile tool, molecular beam epitaxy (MBE) has natural advantages in high-quality heterostructure growth thanks to its ultra-high vacuum (UHV) environment, atomic layer epitaxy accuracy and controllability, instant introduction and control of multiple sources, easy of doping of materials and *in situ* layer-by-layer characterization. As a matter of fact, vdW epitaxy was first demonstrated using MBE process^24^,^25^. Most recently, MBE has also been used to successfully synthesize single-layer and bilayer graphene^26^,^27^,^28^,^29^. In this paper, we report MBE growth of high-quality graphene/h-BN stacked heterostructures on cobalt substrate. Scalable wafer-scale graphene/h-BN films with a misorientation of less than 1° were achieved.

## Experiments

Thermally oxidized Si wafers with a SiO_2_ layer of 300 nm were used as substrates. They were transferred to an E-beam evaporator system for the deposition of a Co film of 400 nm. The wafers were subsequently loaded on to standard 3″ wafer holders and transferred to an MBE system for growth. A Knudsen effusion cell filled with B_2_O_3_ powder (Alfa Aesar, 99.999%) was used as B source. A thermocracker was used to crack acetylene gas (Airgas, 99.999%) as C source. The C source was tuned by either acetylene gas flow or cracker temperature. An electron cyclotron resonance (ECR) system was used to form nitrogen gas plasma (Airgas, 99.9999%) as N atomic source. The N source was tuned by either nitrogen gas flow or ECR magnetron current.

For a typical growth, the substrate is firstly annealed at 800 ~ 850 °C under a hydrogen flow of 10 sccm for a duration of 30 minutes. At the end of substrate surface treatment, the hydrogen gas flow is stopped and substrate temperature is further ramped up to 870 ~ 900 °C for graphene/h-BN heterostructure growth. The thermal cracker temperature is ramped to 1200 °C and 3 sccm acetylene is introduced into the chamber for graphene growth. The growth lasts from 10 s to 1 minute. Subsequently, h-BN growth follows with minimal time gap. B cell temperature is precisely ramped to 900 ~ 1100 °C right before h-BN layer growth. Nitrogen flow rate is 10 sccm and the growth lasts 10 ~ 15 minutes. At the end of h-BN growth, the substrate temperature is slowly cooled down towards room temperature at a rate of 10 °C/min. The slow substrate cooling process suggests that the epitaxy undergoes layer-by-layer growth mode, which is in contrast to fast cooling procedure with much higher cooling rates in the growth of graphene by precipitation of carbon atoms from the metal substrate in some CVD process^30^,^31^.

Raman characterizations were performed using a HORIBA LabRam system equipped with a 50-mW 514-nm green laser. Scanning electron microscopy (SEM) images were acquired using a Philips XL30-FEG system. X-ray photoelectron spectroscopy (XPS) was carried out using a Kratos AXIS ULTRA XPS system equipped with an Al Kα monochromatic X-ray source and a 165 mm mean radius electron energy hemispherical analyzer. Transmission electron microscopy (TEM) images and electron diffraction patterns were acquired using a FEI/Philips CM-30 TEM. Plan-view TEM sample was prepared using direct transfer method. After spin coated with PMMA, the sample was submerged in FeCl_3_ solution to etch away the Co metal layer, which often takes several days. The film was then transferred onto carbon coated Cu TEM grid and treated with acetone and DI water to remove PMMA. Cross sectional TEM sample was prepared using focused ion beam technique. The graphene/h-BN thin film was covered by an Ir layer and further protected by electron-beam and ion-beam deposited Pt layers.

## Results and Discussion

**Figure 1:**
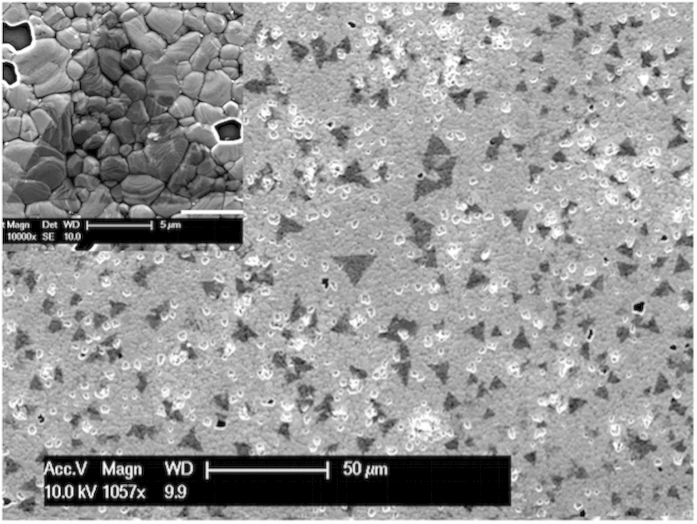
SEM image of graphene/h-BN heterostructure on cobalt substrate (Sample A). Inset is taken under the same condition with larger magnification, and it shows a large triangular h-BN flake of about 20 μm. The background is the rough substrate surface with packed Co grains resulted from heat treatment.

As grown vdW thin films are transparent and thus not visible for bare eye or through optical microscope (Fig. S1, supporting material). As the samples are characterized by SEM, a surface contrast is observed in secondary electron image because secondary electron signals from h-BN and graphene/Co substrate have different intensity. Figure 1 shows an SEM image of a graphene/h-BN sample (Sample A) on which large triangular domains of ~20 μm are observed. Despite the underlying very rough Co substrate surface, which consists of small grains due to heat treatment, wafer-scale graphene has been grown, followed by the formation of the triangular shaped h-BN domains as inferred by both optical microscope imaging and Raman scattering results (Figs S1 and S2 supporting material).

In order to achieve continuous wafer-scale h-BN single domain instead of discrete flakes, fine-tuning of the sample growth condition was performed. Detailed difference in growth condition is summarized in Table 1 of supporting material, in which four samples are listed. By reducing high-temperature substrate treatment duration, graphene layer thickness, and growth temperature for h-BN layer, the resulting sample (Sample B) shows smoother surface morphology under optical microscopy (Fig. S3, supporting material). The shorter growth time and thinner graphene layer clearly improve h-BN layer growth. Sample B (Table 1, supporting material) exhibits the graphene/h-BN film that covers the entire sample surface, and triangular h-BN domains are no longer visible (Fig. S4, supporting material). Although wafer-scale h-BN/graphene heterostructure thin films were also obtained for samples (Samples S1 and S2) grown with the same substrate treatment procedure, higher graphene growth temperature, and longer h-BN growth time as that of Sample A, these films are not as uniform as that of Sample B (Fig. S5, supporting material). Further detailed characterizations were based on Sample B.

**Figure 2:**
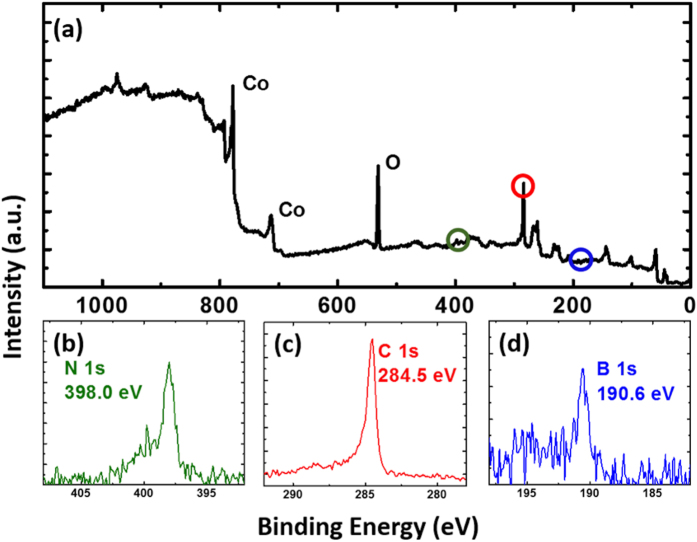
XPS spectra of graphene/h-BN heterostructure on Co substrate (Sample B). (**a**) Survey spectrum, (**b**) N1s peak, (**c**) C1s peak, and (**d**) B1s peak. B1s and N1s are at 190.6 eV, and 398.0 eV, respectively, indicating the existence of h-BN. C1s peak is at 284.5 eV, indicating the presence of graphene.

Figure 2a shows an XPS survey scan spectrum, with the peaks of interest circled. Fine scans were performed in these sites and evident energy peaks for B, C, and N were observed. Figure 2b–d show XPS spectrum of B1s, C1s, and N1s state, respectively. C1s peak is observed at a position of 284.5 eV, which is smaller than environmental C1s peak in XPS, and is closer to the sp^2^ C-C bond at 284.0 eV^32^. This is an indication of graphene. B1s and N1s exhibit an energy position at 398.0 eV and 190.6 eV, respectively, which are typical characteristics of h-BN^33^. Based on integrated peak intensity and corrections, B/N ratio was estimated to be 1.1 from the surface of h-BN, indicating that the growth of h-BN is slightly B-rich.

**Figure 3:**
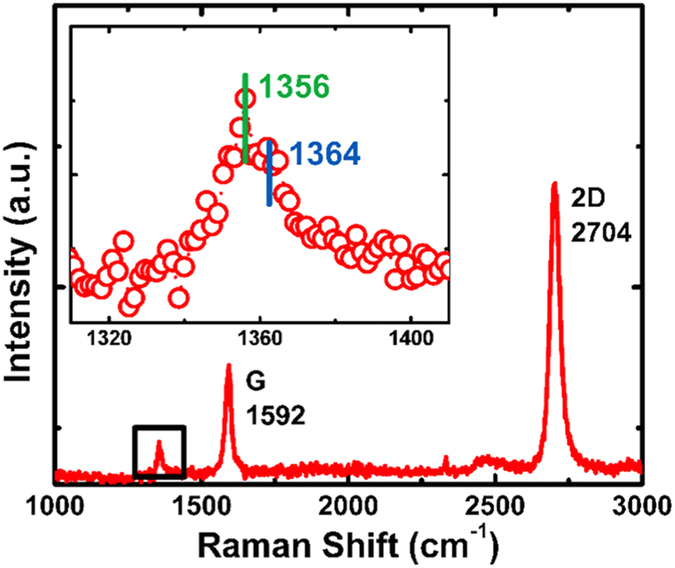
Raman spectrum of graphene/h-BN heterostructure (Sample B). Evident graphene G and 2D peaks are observed, with their intensity ratio indicating 1 ~ 2 layer graphene. The inset is enlarged spectrum in the 1300 ~ 1400 cm^−1^ region. Two peaks are resolved, relating to graphene D mode and E_2g_ optical phonon peak of h-BN, respectively.

Figure 3 shows Raman spectrum of the sample. The G/2D peak ratio of the graphene signals indicates the existence of single-layer/bilayer graphene. The inset is a spectrum near the graphene D peak, which lies at 1356 cm^−1^. The appearance of D peak has been observed in other reports of graphene/h-BN heterostructure^34^. In addition, an h-BN E_2g_ optical phonon peak is evident at 1364 cm^−1^. Analyzing the 2D peak of graphene to detect the mismatch angle of graphene and h-BN has been reported recently^35^. In this experiment, the FWHM of the 2D peak of as grown sample is 38 cm^−1^, which indicates the mismatch angle is below 1°.

**Figure 4:**
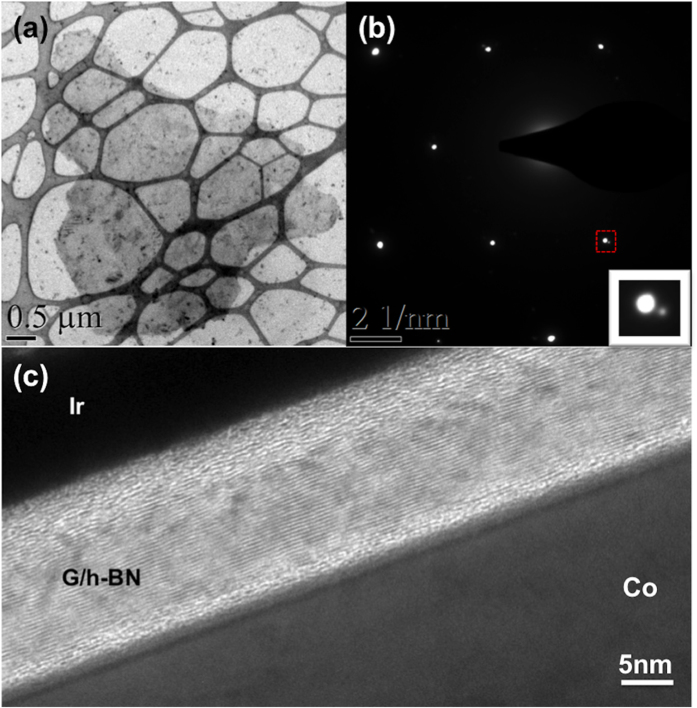
(**a**) Plan-view TEM image of transferred graphene/h-BN heterostructure (Sample B). (**b**) SAED pattern of Sample B. Diffraction patterns with six-fold symmetry are observed. The inset is enlarged image of the red square area marked in Fig. 4(b). Two diffraction spots are observed, revealing the (100) plane distance of 2.13 Å and 2.06 Å, respectively. (**c**) Cross-sectional TEM image of Sample B. The thickness of the heterostructure is about 15 nm.

Figure 4a shows a plan-view TEM image of the transferred graphene/h-BN heterostructure. Continuous thin film is observed with some areas folded. Black spots originate from PMMA residue during film transfer process. Figure 4b shows a selected area electron diffraction (SAED) pattern of the plan-view graphene/h-BN heterostructure thin film. A clear hexagonal set of diffraction spots is observed. A second set, which has weaker intensity and larger diffraction angle, is also seen. The inset shows a zoom-in image of the area marked with a red square, showing clearly two diffraction spots. The two spots correspond to the electron diffraction from (100) planes of h-BN and graphene, respectively. The calculated interplanar spacings corresponding to the two spots are 2.13 Å and 2.06 Å, which match well with expected numbers for h-BN and graphene, respectively. The strong intensity of h-BN diffraction pattern may indicate a well-aligned, multi-layer h-BN. There is a small rotation (<1°) observed between the two sets, similar to other reports on graphene/h-BN heterostructures^36^, which also matches our conclusion from Raman spectrum. Figure 4c shows a cross-sectional TEM image of the heterostructure. Lattice fringes are observed in the ~15nm thick band. The inter-fringe distance is 0.33nm, which is in close agreement with the out-of-plane lattice constants of h-BN and graphene.

## Conclusion

We have demonstrated direct epitaxial growth of h-BN on graphene utilizing MBE. Signature triangular h-BN flakes with sizes as large as 20 μm were observed. Further improvement on the growth condition results in high-quality wafer-scale graphene/h-BN heterostructure single domain film. The epitaxial single/bi-layer graphene/few-layer h-BN structure is further confirmed by XPS, Raman spectroscopy, electron diffraction, and TEM imaging. The misorientation between the graphene and h-BN layers is less than 1°. While MBE synthesis of high-quality graphene/h-BN heterostructures is a critical step toward the realization of 2D electronics, reasonable electronic functionality of heterostructures is of greater concern. In the future work, two- and three-terminal devices will be fabricated and the electrical measurements will be carried out to evaluate their electrical performance.

## Additional Information

**How to cite this article**: Zuo, Z. *et al.* *In-situ* epitaxial growth of graphene/h-BN van der Waals heterostructures by molecular beam epitaxy. *Sci. Rep.* **5**, 14760; doi: 10.1038/srep14760 (2015).

## Supplementary Material

Supplementary Information
