# Supplementary material for: In-situ epitaxial growth of graphene/h-BN van der Waals heterostructures by molecular beam epitaxy
Source: Sci Rep. 2015 Oct 7;5:14760. doi: 10.1038/srep14760 (PMC4595826; doi:10.1038/srep14760)
Supplement: Supplementary Information [file srep14760-s1.pdf]

Supporting material

In-situ epitaxial growth of graphene/h-BN van der Waals heterostructures by molecular beam epitaxy

Zheng Zuo [1, +], Zhongguang Xu [1, +], Renjing Zheng [1, +], Alireza Khanaki [1], Jian-Guo Zheng [2], Jianlin Liu [1,*]

[1] Quantum Structures Laboratory, Department of Electrical and Computer Engineering, University of California, Riverside, California 92521, USA

[2] Irvine Materials Research Institute, University of California, Irvine, CA 92697-2800, USA

*Corresponding E-mail: jianlin@ece.ucr.edu

[+] These authors contributed equally to this work.

Figure S1

Optical microscopy image of graphene/h-BN heterostructure sample with discrete h-BN triangular domains (Sample A)

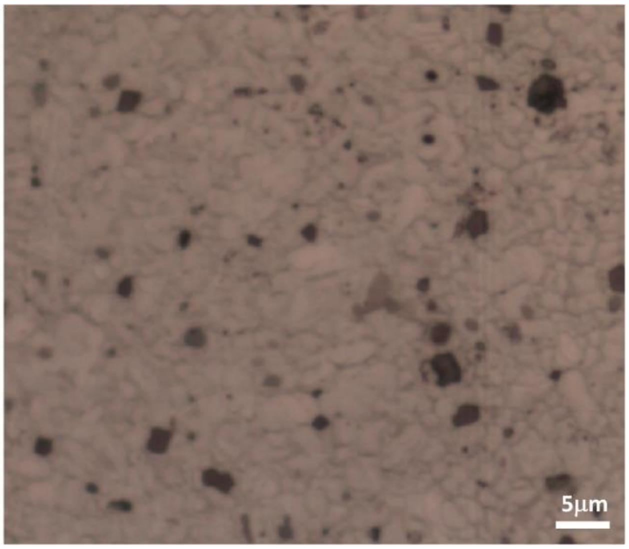

There are some surface imperfections, possibly a result from the high temperature growth. H-BN domains are of large scale and transparent.

Figure S2

Raman spectra of graphene/h-BN sample with discrete h-BN triangular domains (Sample A)

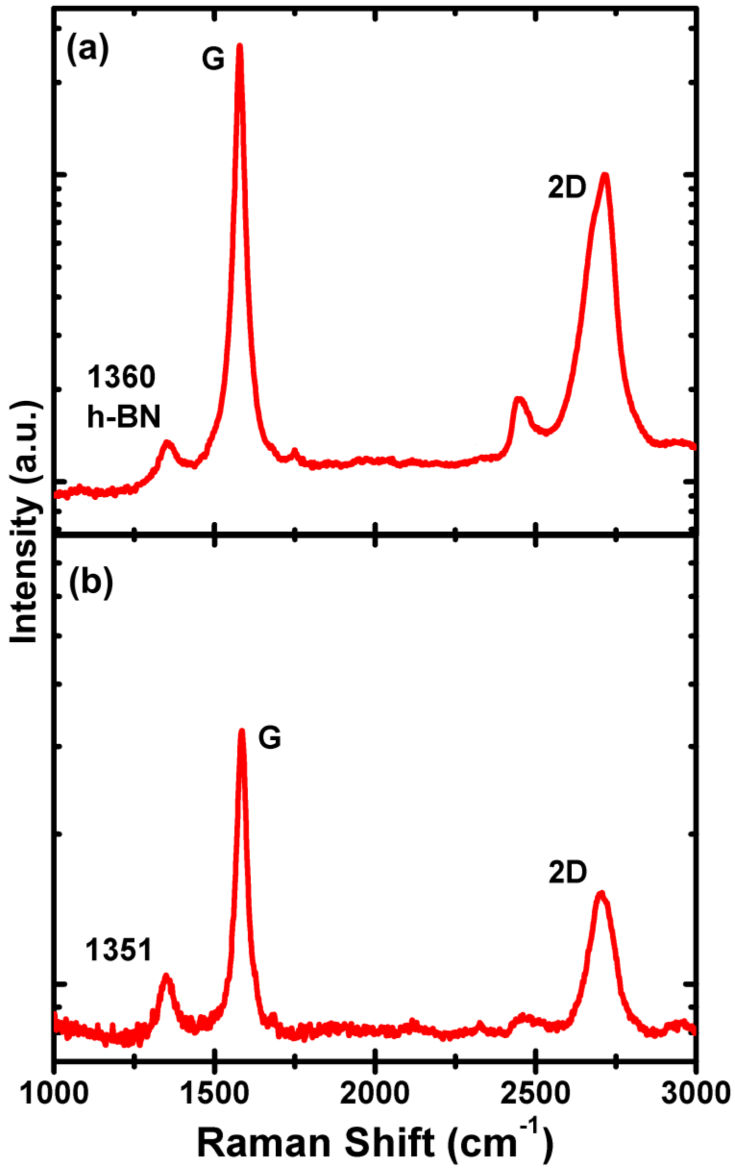

Raman spectra acquired at different locations across the sample surface. Figure S2(a) and S2(b) are typical graphs with S2(a) showing both h-BN E_{2g} optical phonon peak at 1360 cm^{-1} and signature graphene G and 2D peaks at discrete locations, and S2(b) showing only graphene peaks across the entire surface. This result is in close agreement with the SEM result shown in Figure 1 in the text.

Table 1

Comparison of sample growth condition

Step	Parameters	Sample A	Sample S1	Sample S2	Sample B
**Pre-Growth Annealing**	**Temperature**	**800 °C**	**800 °C**	**800 °C**	**850 °C**
	**Duration**	**40 minutes**	**40 minutes**	**40 minutes**	**10 minutes**
**Graphene Growth**	**Substrate temperature**	**800 °C**	**900 °C**	**870 °C**	**850 °C**
	**Gas flow**	**3 sccm**	**3 sccm**	**3 sccm**	**3 sccm**
	**Cracker temperature**	**1200 °C**	**1200 °C**	**1200 °C**	**1000 °C**
	**Growth duration**	**1 minute**	**2 minutes**	**30 s**	**10 s**
**h-BN Growth**	**Substrate temperature**	**900 °C**	**900 °C**	**870 °C**	**850 °C**
	**Boron cell temperature**	**1000 °C**	**1000 °C**	**970 °C**	**950 °C**
	**Nitrogen gas flow**	**10 sccm**	**10 sccm**	**10 sccm**	**10 sccm**
	**Nitrogen ECR current**	**50 mA**	**50 mA**	**50 mA**	**60 mA**
	**Growth duration**	**15 minutes**	**30 minutes**	**30 minutes**	**10 minutes**

Growth condition was fine tuned to reduce heat treatment duration, graphene layer thickness, and h-BN layer coverage from Sample A to Sample B. Sample A is the sample showing triangular domains. For Sample B, graphene was grown by using 50 °C higher substrate temperature and h-BN was grown by using 50 °C lower substrate temperature than Sample A. In this case, both graphene and h-BN were grown at the same substrate temperature of 850 °C, thus the growth time delay between graphene layer and h-BN layer was minimized. Samples S1 and S2 were grown using the same substrate annealing condition as that of Sample A, while their graphene layers were grown at a higher temperature and h-BN layers were grown with a longer time.

Figure S3

Optical microscopy image of graphene/h-BN sample with wafer-scale single domain (Sample B)

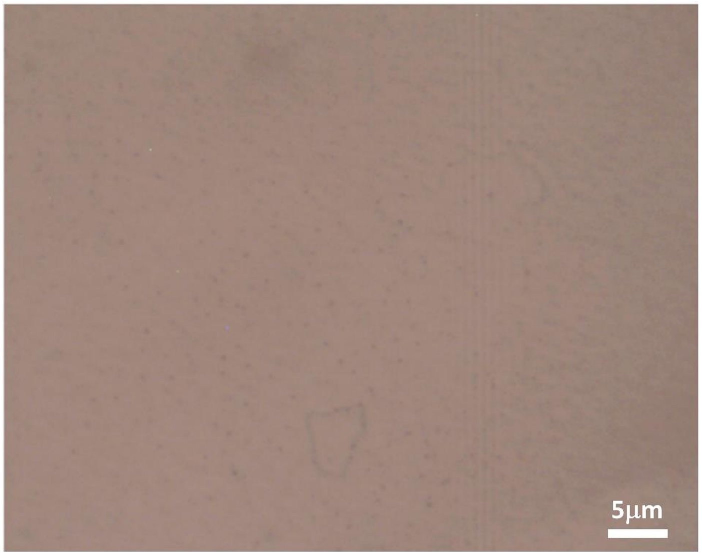

Improved growth condition led to smoother Co substrate surface and wafer-scale graphene/h-BN heterostructure.

Figure S4

SEM image of Sample B with wafer-scale h-BN and graphene coverage

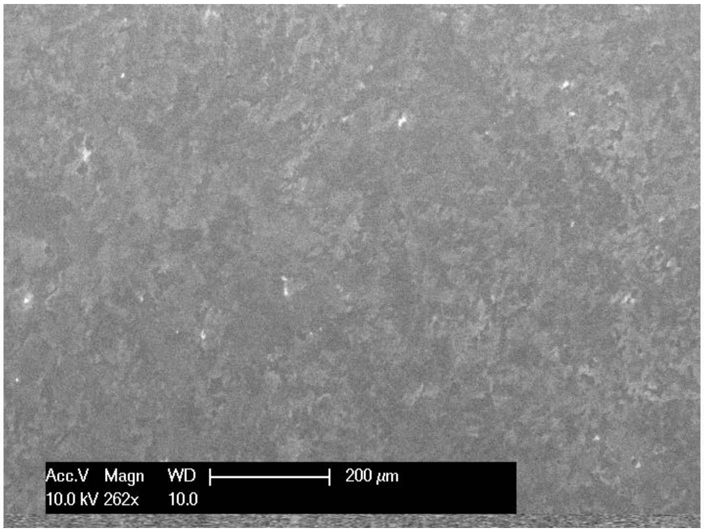

Sample B shows dark contrast film that covers the entire surface, without evident triangular domains.

Figure S5

Optical microscopy images and Raman spectra for Sample S1 (a, b) and Sample S2 (c, d)

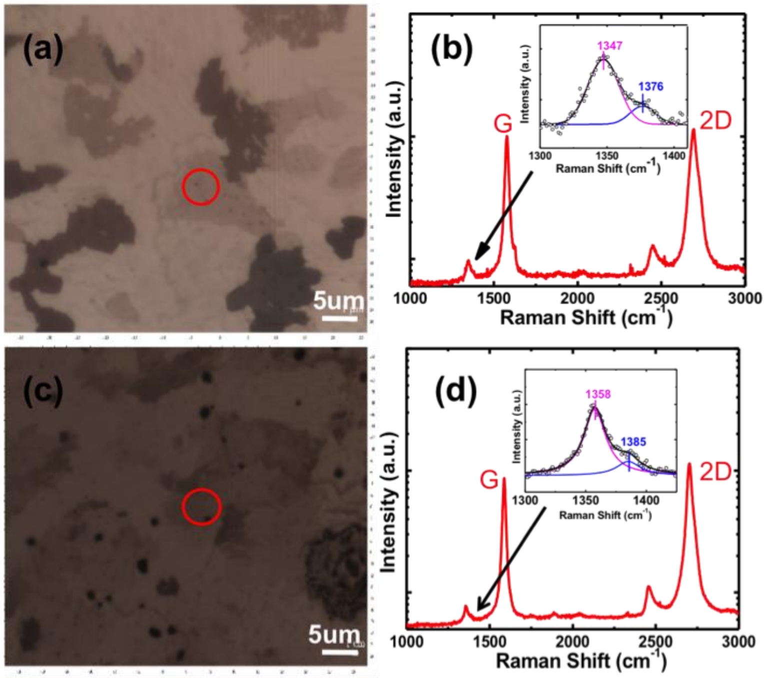

Both Sample S1 and S2 show continuous graphene/h-BN heterostructure films, however they are not as uniform as that of Sample B.
